# Aortic elasticity after aortic coarctation relief: comparison of surgical and interventional therapy by cardiovascular magnetic resonance imaging

**DOI:** 10.1186/s12872-019-01270-w

**Published:** 2019-12-12

**Authors:** Theresa Pieper, Heiner Latus, Dietmar Schranz, Joachim Kreuder, Bettina Reich, Kerstin Gummel, Helge Hudel, Inga Voges

**Affiliations:** 1 Paediatric Heart Centre, University Hospital Giessen & Marburg, Marburg, Germany; 2 Department of Paediatric Cardiology and Congenital Heart Disease, German Heart Centre Munich, München, Germany; 3 Institute for Medical Informatics, Justus-Liebig-University Giessen, Giessen, Germany; 4 Department of Congenital Heart Disease and Paediatric Cardiology, University Hospital Schleswig-Holstein, Arnold-Heller Strasse 3, 24105 Kiel, Germany

**Keywords:** Coarctation of the aorta, Cardiovascular magnetic resonance imaging, Aortic elasticity, Endovascular stent implantation, T1 mapping

## Abstract

**Background:**

Patients after aortic coarctation (CoA) repair show impaired aortic bioelasticity and altered left ventricular (LV) mechanics, predisposing diastolic dysfunction. Our purpose was to assess aortic bioelasticity and LV properties in CoA patients who underwent endovascular stenting or surgery using cardiovascular magnetic resonance (CMR) imaging.

**Methods:**

Fifty CoA patients (20.5 ± 9.5 years) were examined by 3-Tesla CMR. Eighteen patients had previous stent implantation and 32 had surgical repair. We performed volumetric analysis of both ventricles (LV, RV) and left atrium (LA) to measure biventricular volumes, ejection fractions, left atrial (LA) volumes, and functional parameters (LAEF_Passive_, LAEF_Contractile_, LAEF_Reservoir_). Aortic distensibility and pulse wave velocity (PWV) were assessed. Native T1 mapping was applied to examine LV tissue properties. In twelve patients post-contrast T1 mapping was performed.

**Results:**

LV, RV and LA parameters did not differ between the surgical and stent group. There was also no significant difference for aortic distensibility, PWV and T1 relaxation times. Aortic root distensibility correlated negatively with age, BMI, BSA and weight (*p* < 0.001). Native T1 values correlated negatively with age, weight, BSA and BMI (*p* < 0.001). Lower post-contrast T1 values were associated with lower aortic arch distensibility and higher aortic arch PWV (*p* < 0.001).

**Conclusions:**

CoA patients after surgery or stent implantation did not show significant difference of aortic elasticity. Thus, presumably other factors like intrinsic aortic abnormalities might have a greater impact on aortic elasticity than the approach of repair. Interestingly, our data suggest that native T1 values are influenced by demographic characteristics.

## Background

Surgical repair of coarctation of the aorta (CoA) was first performed in 1944 and the techniques have evolved over time with the today preferred surgical method of an end-to-end anastomosis [1]. In addition, there have been significant advances in transcatheter therapies including endovascular stent implantation [2, 3].

Nowadays, early CoA treatment is recommended, and it has been shown that this minimizes the risk of long-term complications such as arterial hypertension [4, 5]. However, more recent reports show, that patients after CoA repair are still at risk for long-term complications, in addition to the aforementioned, in particular increased aortic stiffness and left ventricular (LV) diastolic dysfunction [6–8]. Nevertheless, these studies mainly included patients after surgical treatment. Stent implantation can alter the compliance of the vessel [9], however, data of CoA patients who underwent stent implantation are rare. Babu-Narayan et al. has shown improved aortic distensibility after stenting [10]. Another recent study found no difference in aortic stiffness and endothelial function between CoA patients who were treated with surgery, balloon dilation, or stent implantation [11].

Cardiovascular magnetic resonance (CMR) imaging is commonly used in the long-term follow-up of CoA patients [12]. CMR is typically performed to assess aortic anatomy and ventricular size and function but also enables to measure aortic bioelasticity parameters [8] and to characterise myocardial tissue properties [13]. T1 mapping with calculation of the extracellular volume (ECV) is a newer CMR technique enabling to diagnose diffuse myocardial fibrosis and interstitial myocardial disease [13, 14]. Puntmann et al. have demonstrated a relationship between aortic stiffness, aging and increased interstitial myocardial fibrosis, respectively [15] but detailed data for CoA patients are not available.

We hypothesized that patients who underwent endovascular stent implantation have different aortic elastic and LV functional properties compared to patients who underwent surgical CoA repair. This prospective CMR imaging study aimed to examine aortic elasticity markers, LV functional parameters and LV myocardial tissue properties in patients after endovascular stent implantation and compared them with a group of patients who had surgical CoA repair.

## Methods

### Study population

Fifty patients with isolated CoA (median age 19.3 years, range 4–40 years), who were treated at the Paediatric Heart Center, University Hospital of Giessen and Marburg and who were scheduled for a routine CMR study, were included in this study. The patients were divided into two groups: 1) 32 had surgical CoA repair (29 end-to-end anastomosis, 1 subclavian flap repair, 1 isthmus plastic with equine pericardial patch, 1 Dacron graft) and 2) 18 patients underwent endovascular stent implantation. Eight of them had previous surgical repair (5 end-to-end anastomosis, 1 end-to-side anastomosis, 1 subclavian flap repair, 1 isthmus plastic) but developed re-coarctation requiring further intervention. Balloon angioplasty of the aortic isthmus was performed in 8 patients in the surgical group. Four of them had balloon angioplasty before surgery. In the stent group 10 patients underwent balloon angioplasty. Three of them had balloon angioplasty prior to stent implantation, one patient underwent balloon angioplasty before and after stent implantation and 6 patients underwent balloon angioplasty only after stent implantation.

Patients were excluded, if: a) they had moderate or severe aortic (mean pressure drop ≥25 mmHg) and mitral valve stenosis (mean pressure drop ≥5 mmHg), b) they had moderate or severe aortic (regurgitant fraction ≥20%) and mitral valve insufficiency (regurgitant fraction ≥20%), and c) they presented with a doppler gradient ≥3 m/s in the aortic isthmus and showed a blood pressure gradient ≥20 mmHg between upper and lower extremities. In addition, we excluded patients with other contraindications to CMR.

In one patient, who underwent CMR for clinical reasons, sedation was performed using propofol and midazolam. Electrocardiogram and blood pressure were monitored during all studies using a CMR compatible monitoring system with a cuff placed around the right arm (PrecessTM, Invivo, Florida, USA). Additional oxygen saturation was measured if necessary clinically.

In children (patients < 18 years) blood pressure percentiles were calculated using the fourth report from the National High Blood Pressure Education Program, Working Group on Children and Adolescents from the US National Institutes of Health [16]. In adults (patients ≥18 years) arterial hypertension was defined using the 2013 guidelines from the task force for the management of arterial hypertension of the European Society of Hypertension and the European Society of Cardiology [17].

### CMR image acquisition

All patients underwent 3-Tesla-CMR (Magnetom® Verio, software version syngo, MR B 17, Siemens Healthcare GmbH, Erlangen, Germany) using two 16-channel phased array coils. Axial and sagittal gradient echo cine images were acquired to cover the entire aorta, to measure aortic cross-sectional areas for distensibility assessment and to measure left atrial (LA) volumes [8]. The sequence parameters were as follows: field of view (FOV) 16 to 36 cm, repetition time (TR)/ echo time (TE) 78.3/2.6 ms, flip angle 12°, slice thickness 6 mm, voxel size 1.56 × 1.56 × 6 mm, breath-hold.

For calculation of aortic pulse wave velocity (PWV) in the entire thoracic aorta, phase-contrast cine imaging was applied (parameters: FOV 19 to 40 cm, TR/TE 34.4/2.9 ms, flip angle 25°, slice thickness 5 mm, voxel size 1.72 × 1.72 x5mm, velocity encoding strength 200 to 250 cm/s) [8].

Short axis cine stacks were acquired using a cine steady-state free precession sequence (parameters: TR/TE 47/1.5 ms, flip angle 60°, slice thickness 7 mm, voxel size 1.6 × 1.6 x 7mm, breath-hold).

Native T1 mapping data were collected in basal, midventricular and apical short axis planes with a breath-held modified Look-Locker inversion recovery (MOLLI) sequence (parameters: FOV 306 × 360 mm, TR/TE 4.9/1.2 ms, flip angle 35°, slice thickness 8 mm, voxel size 1.4 × 1.4 × 8 mm). Post contrast T1 mapping was performed 10 min after administration of 0.2 mmol/kg i.v. gadobutrol (Gadovist®, Bayer Healthcare Germany).

### CMR image analysis

All analyses were performed with commercially available software (cmr42; Circle Cardiovascular Imaging, Calgary, Canada). LV and RV endo- and epicardial contours were manually drawn in the short axis stack to measure end-diastolic and end-systolic volumes (LVEDV, LVESV, RVEDV, RVESV) as well as ventricular mass. LV and RV ejection fraction and stroke volume were calculated automatically from the volumes.

Maximal and minimal LA volume (LA_max_, LA_min_) as well as LA volume before atrial contraction (LA_ac_) were measured from transaxial cine stacks as described before [8]. From these measurements additional volumes and functional parameters were generated [18]: LA total emptying volume (LA_totemp_), LA passive emptying volume (LA_passemp_), LA contractile volume (LA_contr_), LA passive emptying function (LAEF_Passive_), LA contractile emptying function (LAEF_Contractile_) and LA reservoir emptying function (LAEF_Reservoir_).

Minimal and maximal aortic cross-sectional areas were measured at six positions from CMR cine images: aortic root, ascending aorta (AAo), transverse aortic arch, aortic isthmus, proximal descending aorta (DAo) and DAo above the diaphragm. Aortic distensibility was calculated using the following formula [19]:

$$ \mathrm{Distensibility}\ \left(1{0}^{-3}\;{\mathrm{mmHg}}^{-1}\right)=\left({\mathrm{A}}_{\mathrm{max}}-{\mathrm{A}}_{\mathrm{min}}\right)/\left[{\mathrm{A}}_{\mathrm{min}}\mathrm{x}\ \left({\mathrm{P}}_{\mathrm{max}}-{\mathrm{P}}_{\mathrm{min}}\right)\right] $$

Areas of maximal distension (A_max_) were measured in systole, areas of minimal distension (A_min_) in diastole. Systolic and diastolic blood pressure (P_max_ and P_min_) measurements were taken as described above.

PWV for the entire thoracic aorta was measured as described previously by one of the authors [20].

T1 times were measured in the interventricular septum and within the entire LV myocardium on all three slices [14, 21]. Post-contrast T1 relaxation times were measured in 12 patients using the same postprocessing methods. Extracellular volume fraction (ECV) was calculated as described before [14, 21].

### Statistical analysis

Statistical analysis was performed using SPSS Statistics (IBM Corp. released 2016. IBM® SPSS® Statistics for Mac. Version 24.0. Armonk, NY: IBM Corp.). To determine if a variable was normally distributed, the Kolmogorow-Smirnow-Test with modification after Lilliefors was applied. All normally distributed data was shown appropriately as mean ± standard deviation, otherwise they were presented as median with range. Data was adequately evaluated according to their distribution by T-test or Mann-Whitney-U-Test. Adjustments for multiple testing were performed and the significant *p*-value was reduced to 0.005.

Correlations between variables were analysed with Pearson’s correlation for normally distributed variables and Spearman’s rho for non-normally distributed data. *P*-values of < 0.05 were indicated as statistically significant.

## Results

Parts of this work were presented at 52nd Annual Meeting of the Association for European Paediatric and Congenital Cardiology (AEPC) Megaron Athens International Conference Centre, Athens, Greece, May 9–12, 2018 [22].

### Patients

Characteristics of the stent and surgical groups did not differ significantly apart from age at repair (Table 1). Twenty-eight patients (56%) had a bicuspid aortic valve, 22 (79%) of them had mild aortic regurgitation and 15 (54%) had mild aortic stenosis. All other patients (*n* = 6) with bicuspid aortic valve had normal blood flow profiles. Twenty-two patients (44%) had a trileaflet aortic valve. One patient of them had mild aortic valve stenosis and four patients had mild aortic regurgitation.

**Table 1:** Group characteristics

Measurements	All, *N* = 50	Surgery, *N* = 32	Stent, *N* = 18	*p*-value
Age at treatment (years)	1.5 (0.0–24.5)	0.4 (0.0–12.6)	6.7 (0.0–24.5)	0.04
Years after treatment	13.2 (0.7–38.7)	17.2 (4–35)	6.6 (0.7–38.7)	0.05
Age at CMR (years)	20.5 ± 9.5	20.5 ± 9.7	20.5 ± 9.5	0.99
Weight (kg)	60.7 ± 22.7	59.9 ± 24.0	61.9 ± 20.8	0.8
Height (cm)	166.0 (111–195)	167.0 (111–195)	166.0 (118–190)	0.86
BMI (kg/m^2^)	21.6 ± 5.2	21.4 ± 4.7	22.0 ± 6.3	0.73
BSA (m^2^)	1.7 ± 0.4	1.6 ± 0.4	1.7 ± 0.3	0.7
Diastolic BP (mmHg)	63.1 ± 9.4	62.7 ± 9.4	64.0 ± 9.7	0.65
Systolic BP (mmHg)	117.0 (90.0–165.0)	115.5 (90.0–165.0)	118.5 (96.0–147.0)	0.07
Pulse pressure (mmHg)	55.5 ± 12.5	53.8 ± 12.9	58.5 ± 11.6	0.20
Heart rate (b/min)	74.0 (48.0–110.0)	74.0 (48.0–110.0)	71.5(51.0–91.0)	0.27
Medication				
- ACE inhibitor (n)	2	1	1	
- ARB ((n)	16	9	7	
- Betablocker (n)	12	6	6	
- ASA (n)	1	0	1	

*ACE* Angiotensin converting enzyme inhibitor, *ARB* Angiotensin receptor blocker, *ASA* Acetylsalicylic acid, *BMI* Body mass index, *BSA* Body surface area, *CMR* Cardiovascular magnetic resonance, *BP* Blood pressure

Four patients in the surgical group had a ventricular septal defect and underwent closure of the defect at the time of CoA repair. Five patients had mild mitral valve regurgitation and one had mild increased forward flow across the mitral valve.

Twenty-five patients had a diagnosis of arterial hypertension with 23 of them on antihypertensive treatment (Table 1). Blood pressure measurements at the time of CMR demonstrated elevated systolic blood pressure in five patients. Three of those patients were treated for arterial hypertension. The arm-leg systolic blood pressure difference was for all patients 4.6 mmHg and there was no difference between the two groups.

### Aortic dimensions, distensibility and PWV

Aortic cross-sectional areas at all six positions did not differ between patients who had surgical CoA repair and patients who underwent stent implantation (aortic root: 354 mm^2^/m^2^ vs. 399 mm^2^/m^2^; AAo: 301 mm^2^/m^2^ vs. 294 mm^2^/m^2^; aortic arch: 177 mm^2^/m^2^ vs. 178 mm^2^/m^2^, aortic isthmus: 149 mm^2^/m^2^ vs. 155 mm^2^/m^2^; proximal DAo: 159 mm^2^/m^2^ vs. 149 mm^2^/m^2^; DAo at diaphragm: 159 mm^2^/m^2^ vs. 146 mm^2^/m^2^; *p* = 0.26–0.98).

**Fig. 1:**
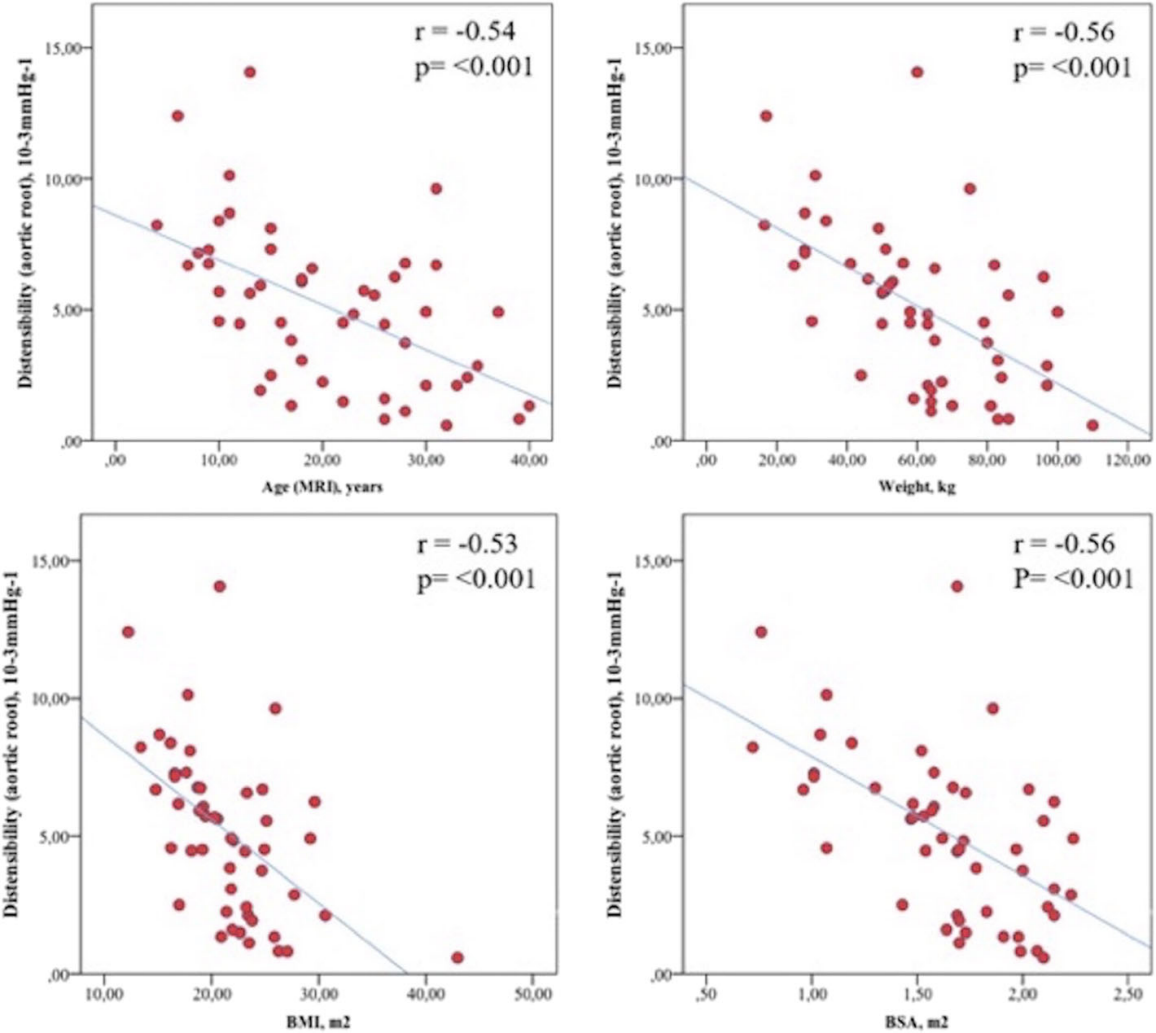
Relationship between aortic root distensibility and anthropometric characteristics

There were also no significant differences in thoracic aortic distensibility as well as aortic arch and DAo PWV between the two study groups (Table 2). Aortic root distensibility correlated negatively with age (r = − 0.5), body mass index (r = − 0.5), body surface area (r = − 0.6), and weight (r = − 0.6), respectively (all *p* < 0.001, Fig. 1).

**Table 2:** Comparison of CMR measurements in patients with surgical repair and endovascular stent implantation

CMR data	Surgery, *N* = 32	Stent, *N* = 18	*p*-value	CI (95)^a^
LVEDVi (ml/m^2^)	73.5 (55.4–102.3)	78.7 (60.8–129.6)	0.04	− 13.8 - -0.06
LVESVi (ml/m^2^)	26.3 (18.2–37.6)	26.5 (20.0–51.7)	0.41	−6.62 – 2.22
LVSVi (ml/m^2^)	46.7 (35.1–70.6)	51.7 (36.6–77.9)	0.03	− 9.42 - -0.98
LVEF (%)	64.3 (55.1–75.9)	65.9 (56.1–70.7)	0.64	−3.62 – 2.63
LVmass index (g/m^2^)	57.7 (31.8–75.8)	60.2 (28.1–116.5)	0.23	−17.6 – 3.19
RVEDVi (ml/m^2^)	74.0 (51.7–109.8)	75.8 (55.4–111.4)	0.59	−8.73 – 5.62
RVESVi (ml/m^2^)	28.7 (17.1–55.4)	30.9 (19.3–48.2)	0.92	− 5.05 – 5.65
RVSVi (ml/m^2^)	43.4 (26.5–68.4)	47.3 (9.8–68.5)	0.21	− 7.82 – 3.15
RVEF (%)	60.9 (34.3–69.9)	61.3 (16.9–73.3)	0.59	−5.63 – 3.41
RVmass index (g/m^2^)	22.1 (15.1–59.1)	24.9 (14.0–51.2)	0.51	− 5.09 – 2.67
LA_max_ (ml/m^2^)	30.8 ± 8.7	35.5 ± 7.2	0.06	−9.61 – 0.26
LA_min_ (ml/m^2^)	17.0 ± 6.0	19.0 ± 5.7	0.27	−5.52 – 1.60
LA_ac_ (ml/m^2^)	21.8 ± 6.4	22.9 ± 5.1	0.54	−4,70 – 2.49
LA_totemp_ (ml/m^2^)	13.8 ± 5.5	16.5 ± 5.0	0.1	− 5.93 – 0.50
LA_passemp_ (ml/m^2^)	9.0 ± 4.1	12.5 ± 3.9	0.01	−6.04 – − 1.09
LA_contractile_ (ml/m^2^)	4.8 ± 2.9	4.0 ± 4.6	0.43	−1.28 – 2.98
LAEF_reserve_ (%)	43.9 ± 12.7	46.6 ± 12.3	0.48	− 10.3 – 4.89
LAEF_passiv_ (%)	28.2 ± 10.6	35.2 ± 7.5	0.02	−12.8 – − 1.16
LAEF_contractile_ (%)	47.6 ± 20.7	48.1 ± 18.3	0.93	− 12.6 – 11.51
Distensibility (10^− 3^ mmHg^− 1^)				
- Aortic root	4.9 ± 2.6	5.4 ± 3.7	0.64	−2.23 – 1.37
- AAo	6.8 ± 4.7	6.0 ± 3.2	0.51	−1.68 – 3.33
- Aortic arch	6.0 ± 3.0	6.2 ± 2.7	0.80	−1.92 – 1.49
- Isthmus	5.1 ± 3.3	4.8 ± 2.6	0.84	− 1.63 – 1.99
- Proximal DAo	5.0 ± 2.5	5.0 ± 2.1	0.89	− 1.31 – 1.50
- DAo at diaphragm	7.4 ± 3.9	6.3 ± 2.5	0.28	− 0.92 – 3.13
PWV (m/s)^2^				
- Aortic arch	3.8 (2.0–10.0)	4.2 (1.9–7.7)	0.63	
- DAo	4.4 (2.5–107.3)	4.4 (3.3–14.0)	0.34	

Values are mean ± standard deviation or median with range. *P* values of < 0.005 were indicated as statistically significant

*AAo* Ascending aorta, *CI* Confidence interval, *Dao* Descending aorta, *LV* Left ventricle, *LVEF* Left ventricular ejection fraction, *LVSVi* Left ventricular stroke volume index, *LVEDVi* Left ventricular end-diastolic volume index, *LVESVi* Left ventricular end-systolic volume index, *LA*_*max*_ Maximal left atrial volume, *LA*_*min*_ Minimal left atrial volume, *LA*_*ac*_ left atrial volume just before atrial contraction, *LA*_*contr*_ LA contractile volume, *LAEF*_*Contractile*_ Left atrial contractile emptying function, *LAEF*_*Passive*_ Left atrial passive emptying function, *LAEF*_*Reservoir*_ Left atrial reservoir emptying function, *LA*_*passemp*_ LA passive emptying volume, *PWV* Pulse wave velocity, *LA*_*totemp*_ LA total emptying volume, *RV* Right ventricle

^a^Confidence intervals are based on the Hodges-Lehman method [23]

Compared to own normal values for patients until 30 years of age [24], in 20% (*n* = 8) ascending aortic distensibility was below the 5th centile. Distensibility of the DAo at diaphragm level was below the 5th centile in 7 patients (17%). Distensibility of the aortic arch was below the 5th centile in 17 patients (41%). In 32% of patients (*n* = 13) aortic arch PWV was above the 95th centile for healthy controls.

Measurements of aortic diameters were found to be substantially reproducible in a previous study from one of our authors [20]. Furthermore, excellent interobserver correlation was found for calculation of pulse wave velocities by another group [25].

### LV systolic and diastolic function

LVEDV and LVSV were lower in the surgical group compared to the stent group (Table 2). There was no significant difference between both groups for LVESV, LVEF, LVmass, RVEDV, RVESV, RVEF and RVmass (Table 2).

LA volumes were lower in patients who underwent surgery. However, after correction of *p*-values after multiple comparisons testing there was no statistical difference between the study groups (Table 2). LA_max_ trended lower in CoA patients after surgical repair. Similarly, there were no significant differences in LA functional parameters between the study groups (Table 2).

No correlations were found between ventricular functional and aortic elasticity (distensibility, PWV) parameters.

### T1 mapping

**Fig. 2:**
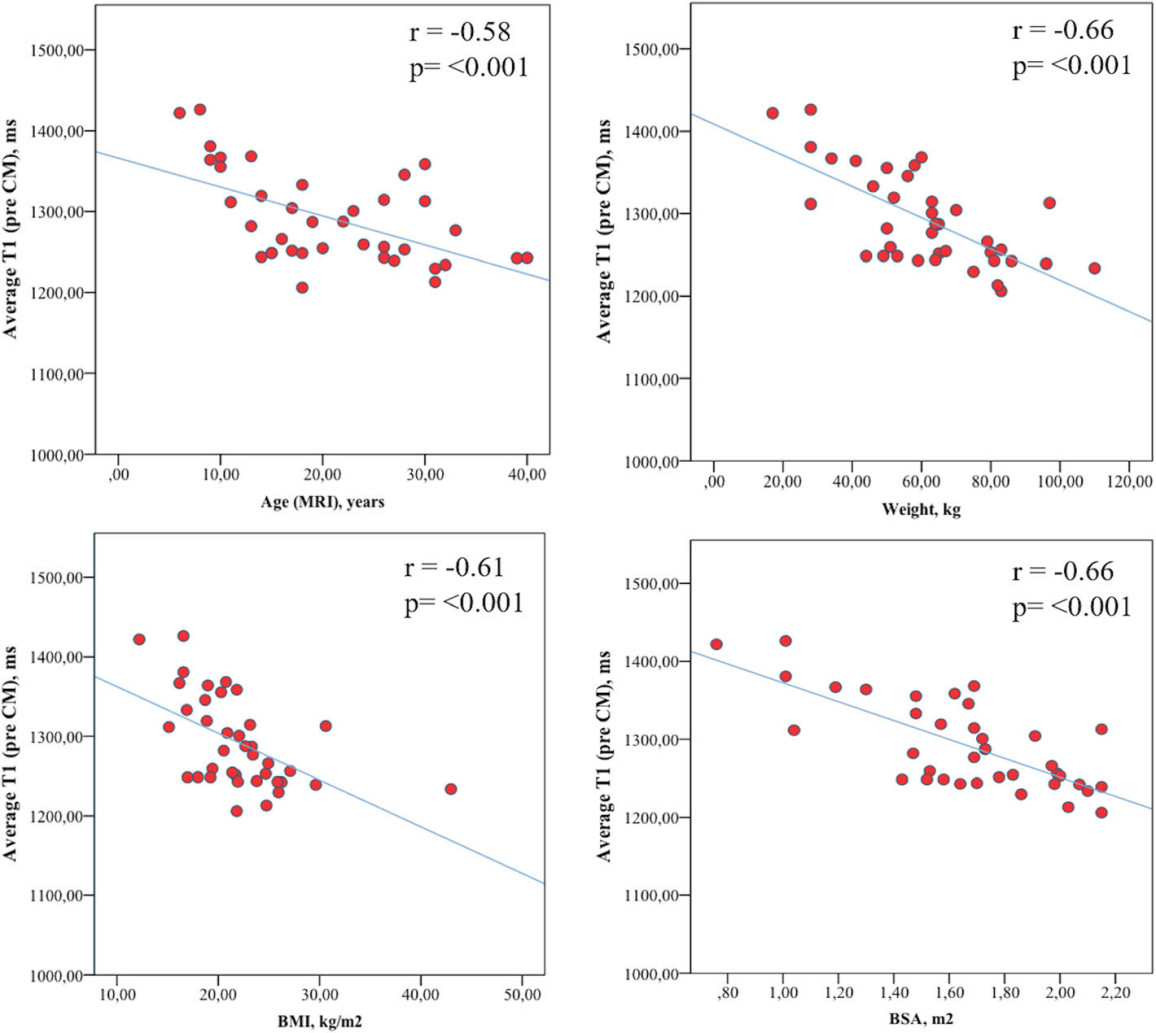
Relationship between native T1 values and anthropometric characteristics

Average native T1 values correlated significantly with age, weight, BMI and BSA (*p* < 0.001, Fig. 2) and post-contrast T1 times correlated with distensibility and PWV (distensibility, r = 0.66, *p* < 0.05; PWV, r = − 0.71; *p* < 0.05).

There were no significant differences for native average and native T1 values per slice as well as mid-septal T1 values between both study groups (Table 3).

**Table 3:** Native and postcontrast T1 relaxation times (in ms)

T1 times	Surgery, *N* = 32	Stent, *N* = 18	*p*-value	CI (95)^a^
Native T1 values				
- Mid-septum	1238.0 (1172.1–1301.7)	1240.9 (828.5–1317.2)	0.94	− 32.8 – 29.9
Average native T1	1262.5 (1206.1–1426.5)	1284.4 (1213.1–1422.1)	0.85	−54.17 – 34.93
Post-contrast T1 values				
- Mid-septum	393.4 (285.5–454.3)	441.1 (311.1–597.0)	0.18	− 174 – 28.3
Average post-contrast T1	390.3 (244.5–443.4)	430.5 (311.1–598.7)	0.13	− 192 – 34.5
ECV (%)	39.8 (36.2–63.1)	33.2 (25.0–41.1)	0.41	−44.0 – 43.7

Values are median with range. *P* values of < 0.005 were indicated as statistically significant

*CI* Confidence interval, *ECV* Extracellular volume fraction

^a^Confidence intervals are based on the Hodges-Lehman method [23]

Post-contrast T1 times and ECV were measured only in a subgroup of patients (*n* = 12). There was no difference for post-contrast T1 times and ECV between the surgical and stent group (Table 3).

## Discussion

Impaired aortic bioelasticity and altered LV mechanical properties have been found in adults and even children after surgical CoA repair, but only few data have been collected so far for CoA patients who underwent endovascular stent implantation. This study compared thoracic aortic elasticity and LV functional parameters as well as LV myocardial T1 times between CoA patients that were treated either by surgery or catheter intervention. Our data did not demonstrate differences for aortic elasticity, LV function and myocardial T1 times. Our study also adds to the current literature that native T1 times in children and young adults are associated with demographic parameters.

### Aortic dimensions, distensibility and PWV

Our study did not show differences in aortic dimensions, aortic distensibility and aortic PWV between the two study groups. Compared to healthy controls, many of our patients below the age of 31 years had reduced distensibility at the aortic isthmus (41%) and increased aortic arch PWV (32%).

Previous studies have shown that CoA patients have reduced aortic bioelasticity and impaired LV function and evidence exists that even after successful repair late complications are common. Vogt et al. reported increased aortic stiffness and reduced aortic distensibility in patients after surgical repair, which remained unchanged during follow-up after surgery; others found impaired endothelial dysfunction [26, 27]. CMR studies after surgical CoA repair showed that even normotensive patients have an increased aortic stiffness and that this is associated with increased LV mass [8, 25, 28]. Our results are comparable with a recently published study which did not show significant differences for aortic stiffness parameters between CoA who were either treated with surgery, balloon dilatation or stent implantation [11]. Babu-Narayan et al., however, showed that adult patients after endovascular stenting have an increased aortic distensibility and they also showed reduced blood pressure indices, improved LVEF and reduced LV mass index [10]. The improved elastic properties after stent implantation could possibly be explained by the effect that stenting reduces the risk of intima hyperplasia and thrombosis [3, 28, 29]. On the other hand, Eicken et al. demonstrated that arterial hypertension persists in many patients after CoA stenting and they discussed that this might be caused by impaired aortic elastic properties [30]. Furthermore, uncoated stents may lead to further re-stenosis while stents with coating possibly support the development of aneurysms. The latter was not assessed in this study and the pathomechanism how endovascular stent implantation can change aortic tissue is not entirely clear yet [3].

In our study, patients who were treated with endovascular stent implantation were older at the time of treatment which can be explained by the fact that stent implantation is normally not the first-line treatment for CoA patients in early infancy. This is due to the fact that re-coarctation requiring interventional re-dilatation of the stent is common [30].

It has been shown that it is important treating CoA patients early after diagnosis to reduce long-term complications [5, 31]. Regarding impaired aortic elasticity, our data however seems to illustrate that possibly there is no difference in aortic elasticity between surgical repair and endovascular stenting at this time of data acquisition. We speculate that the treatment method has less influence on aortic elasticity and that intrinsic aortic wall abnormalities might be mainly responsible for impaired aortic elasticity [32].

### LV function

No significant differences between the study groups for LV systolic and diastolic functional parameters were found.

Using echocardiography, other groups have shown that LV diastolic dysfunction is common after CoA repair in both hypertensive and normotensive patient and may be related to chronically increased aortic stiffness [6, 7]. Voges et al. demonstrated that reduced LA functional parameters correlate with increased aortic arch stiffness in repaired CoA patients and that arterial stiffness is not limited to the aorta suggesting that CoA is a systemic disease [8].

In this study we compared two different treatment groups. Patients who underwent stent implantation were older at the time of intervention and had a shorter follow-up duration. Furthermore, 8 patients who underwent stent implantation had prior surgical repair. Although we did not find a relationship between age at the time of intervention, these factors could have impacted on the LV functional and aortic elasticity results because of longer exposure to increased LV afterload and higher incidence of re-coarctation.

Overall, the results from the current study, however, suggest that CoA treatment techniques are probably less important for the reported LV functional impairment and thereby support the theory that systemic vascular changes might have a greater impact.

### T1 mapping

Myocardial T1 mapping is a technique used for detection of structural changes in the myocardial interstitium. In our study, we found strong correlations between native T1 times age, weight, BMI and BSA (Fig. 2) and post-contrast T1 values correlated with aortic arch distensibility and PWV, respectively.

These findings are conflicting. A recent study by Roy et al. at 3 T, found that T1 increases with age in males but not in females [33]. Knobelsdorrf-Brenkendorff et al. showed that ageing is associated with decreasing native T1 values at 3 T [34]. Both studies only included adults and data for paediatric patients are lacking. Kato et al. performed T1 mapping in paediatric single ventricle patients and showed similarly to our 3 T study that T1 times correlate inversely with age and body weight [35]. The different results of our study might be partially explained by age. We examined a young cohort of patients and Roy et al. [33] included only adult patients with an age range of 20–90 years. Although speculative, it might also be possible that CoA patients have different inherent myocardial tissue characteristics compared to the normal population. However, the influence of age and repaired CoA on native T1 mapping data remains debated and further studies also in CoA patients are needed.

The association between post-contrast T1 values and aortic elasticity parameters in a small subgroup of patients might indicate that increased aortic stiffness has an adverse impact on myocardial tissue characteristics. Puntman et al. performed native T1 mapping and assessment of PWV in patients with dilated cardiomyopathy and healthy controls. They found that PWV is associated with native T1 in the presence of dilated cardiomyopathy [33]. In contrary to them, we did not detect a relationship between native T1 and aortic elasticity parameters. However, we investigated a younger cohort of patients with different disease and therefore our results might not be comparable.

### Limitations

This is a single centre study and the number of patients is therefore limited. In addition, the number of patients in the subgroups was not similar.

Patients who were treated with endovascular stent implantation were older at the time of treatment. Nevertheless, we did not find a correlation between age at treatment and aortic elasticity and LV functional parameters, respectively. Hemodynamic data prior to surgery or intervention for CoA were not available for a significant number of patients, in particular those who underwent surgical CoA repair many years ago. Therefore, we were unable to compare this baseline information with CMR measurements.

Eight patients of the stent group had prior surgical repair, and this might have affected the study results.

Patients with signs of re-CoA or residual CoA were excluded from the study to eliminate additional factors that can have an additional impact on the CMR measurements of aortic bioelasticity, LV functional properties and LV myocardial tissue properties, but exclusion of this patient subgroup might have also influenced the study results.

Reliability of measurements was not assessed in this study. However, interobserver reliability was found to be good for aortic measurements and PWV in previous studies [20, 25]. We did not exclude hypertensive patients. However, the distribution of patients with a known diagnosis of arterial hypertension was similar between both groups.

## Conclusion

After surgical repair or endovascular stent implantation CoA patients without clinically relevant re-coarctation did not show significant difference of aortic elasticity. It can be assumed that other factors like intrinsic aortic wall abnormalities might have a greater impact on aortic wall elasticity than the approach of repair. Interestingly, our data suggest that native T1 values are influenced by age, weight, BSA and BMI.

## Data Availability

The datasets used and/or analysed during the current study are available from the corresponding author on reasonable request.

## Abbreviations

AAo: Ascending aorta
BMI: Body mass index
BSA: Body surface area
CMR: Cardiovascular magnetic resonance imaging
CoA: Coarctation of the aorta
DAo: Descending aorta
ECV: Extracellular volume
EDV: End-diastolic volume
ESV: End-systolic volume
LA: Left atrium
LV: Left ventricle
PWV: Pulse wave velocity
RV: Right ventricle
